# *TP53* Abnormalities Are Underlying the Poor Outcome Associated with Chromothripsis in Chronic Lymphocytic Leukemia Patients with Complex Karyotype

**DOI:** 10.3390/cancers14153715

**Published:** 2022-07-29

**Authors:** Silvia Ramos-Campoy, Anna Puiggros, Joanna Kamaso, Sílvia Beà, Sandrine Bougeon, María José Larráyoz, Dolors Costa, Helen Parker, Gian Matteo Rigolin, María Laura Blanco, Rosa Collado, Idoya Ancín, Rocío Salgado, Marco A. Moro-García, Tycho Baumann, Eva Gimeno, Carol Moreno, Marta Salido, Xavier Calvo, María José Calasanz, Antonio Cuneo, Florence Nguyen-Khac, David Oscier, Claudia Haferlach, Jonathan C. Strefford, Jacqueline Schoumans, Blanca Espinet

**Affiliations:** 1 Molecular Cytogenetics and Hematological Cytology Laboratories, Pathology Department, Hospital del Mar, 08003 Barcelona, Spain; sramosca10@alumnes.ub.edu (S.R.-C.); jkamaso@imim.es (J.K.); egimenov@psmar.cat (E.G.); msalido@psmar.cat (M.S.); xcalvo@psmar.cat (X.C.); 2 Translational Research on Hematological Neoplasms Group, Cancer Research Program, Institut Hospital del Mar d’Investigacions Mèdiques (IMIM), 08003 Barcelona, Spain; 3 Hematopathology Section, Department of Pathology, Hospital Clínic, Institut d’Investigacions Biomèdiques August Pi i Sunyer (IDIBAPS), University of Barcelona, 08036 Barcelona, Spain; sbea@clinic.cat (S.B.); dcosta@clinic.cat (D.C.); tychostephan.baumann@salud.madrid.org (T.B.); 4 Centro de Investigación Biomédica en Red de Cáncer (CIBERONC), 28029 Madrid, Spain; 5 Oncogenomic Laboratory, Hematology Service, Lausanne University Hospital, 1011 Lausanne, Switzerland; sandrine.bougeon@chuv.ch (S.B.); jacqueline.schoumans@chuv.ch (J.S.); 6 Cytogenetics and Hematological Genetics Services, Department of Genetics, University of Navarra, 31008 Pamplona, Spain; mjlarra@unav.es (M.J.L.); mjcal@unav.es (M.J.C.); 7 Cancer Sciences, Faculty of Medicine, University of Southampton, Southampton SO16 6YD, UK; h.parker@soton.ac.uk (H.P.); jcs@soton.ac.uk (J.C.S.); 8 Hematology Section, St. Anna University Hospital, 44121 Ferrara, Italy; rglgmt@unife.it (G.M.R.); cut@unife.it (A.C.); 9 Department of Hematology, Hospital de la Santa Creu I Sant Pau, 08041 Barcelona, Spain; mlblanco@santpau.cat (M.L.B.); cmorenoa@santpau.cat (C.M.); 10 Department of Hematology, Consorcio Hospital General Universitario, 46014 Valencia, Spain; collado_ros@gva.es; 11 Department of Hematology and Hemotherapy, Hospital Universitario Cruces, 48903 Bilbao, Spain; idoyamaria.ancinarteaga@osakidetza.eus; 12 Cytogenetics Laboratory, Hematology Department, Fundación Jiménez Díaz, 28040 Madrid, Spain; rocio.salgado@quironsalud.es; 13 Laboratory Medicine Department, Hospital Universitario Central de Asturias, 33011 Oviedo, Spain; marcoantonio.moro@sespa.es; 14 Applied Clinical Research in Hematological Malignances, Cancer Research Program, Institut Hospital del Mar d’Investigacions Mèdiques (IMIM), 08003 Barcelona, Spain; 15 Sorbonne University, Hematology Department, Hôpital Pitié-Salpêtrière, APHP, INSERM U1138, 75013 Paris, France; florence.nguyen-khac@aphp.fr; 16 Department of Molecular Pathology, Royal Bournemouth Hospital, Bournemouth BH7 7DW, UK; david.oscier@sky.com; 17 MLL Munich Leukemia Laboratory, 81377 Munich, Germany; claudia.haferlach@mll.com

**Keywords:** chronic lymphocytic leukemia, genomic complexity, chromothripsis, *TP53*, genomic microarrays, optical genome mapping

## Abstract

**Simple Summary:**

Chromothripsis, a genomic event that generates massive chromosomal rearrangements, has been described in 1–3% of CLL patients and is associated with poor prognostic factors (e.g., *TP53* abnormalities and genomic complexity). However, previous studies have not assessed its role in CLL patients with complex karyotypes. Herein, we aimed to describe the genetic characteristics of 33 CLL patients with high genomic complexity and chromothripsis. Moreover, we analyzed the clinical impact of chromothripsis, comparing these patients against a cohort of 129 patients with complex karyotypes not presenting this catastrophic event. Nine cases were also assessed via the novel cytogenomic methodology known as optical genome mapping. We confirmed that this phenomenon is heterogeneous and associated with a shorter time to first treatment. Nonetheless, our findings suggested that *TP53* abnormalities, rather than chromothripsis itself, underlie the dismal outcome.

**Abstract:**

Chromothripsis (cth) has been associated with a dismal outcome and poor prognosis factors in patients with chronic lymphocytic leukemia (CLL). Despite being correlated with high genome instability, previous studies have not assessed the role of cth in the context of genomic complexity. Herein, we analyzed a cohort of 33 CLL patients with cth and compared them against a cohort of 129 non-cth cases with complex karyotypes. Nine cth cases were analyzed using optical genome mapping (OGM). Patterns detected by genomic microarrays were compared and the prognostic value of cth was analyzed. Cth was distributed throughout the genome, with chromosomes 3, 6 and 13 being those most frequently affected. OGM detected 88.1% of the previously known copy number alterations and several additional cth-related rearrangements (median: 9, range: 3–26). Two patterns were identified: one with rearrangements clustered in the region with cth (3/9) and the other involving both chromothriptic and non-chromothriptic chromosomes (6/9). Cases with cth showed a shorter time to first treatment (TTFT) than non-cth patients (median TTFT: 2 m vs. 15 m; *p* = 0.013). However, when stratifying patients based on *TP53* status, cth did not affect TTFT. Only *TP53* maintained its significance in the multivariate analysis for TTFT, including cth and genome complexity defined by genomic microarrays (HR: 1.60; *p* = 0.029). Our findings suggest that *TP53* abnormalities, rather than cth itself, underlie the poor prognosis observed in this subset.

## 1. Introduction

The therapeutic landscape for patients with chronic lymphocytic leukemia (CLL) has expanded with the emergence of new targeted agents. In this context, genomic complexity has become increasingly important due to its controversial role as a predictor of response to therapy. It has been associated with shorter survival and worse response rates in patients treated with standard chemoimmunotherapy [1,2,3,4], yet its role in patients receiving new treatment modalities is still not fully established. In the initial trials performed with BTK and BCL2 inhibitors (i.e., Ibrutinib, Acalabrutinib, Venetoclax), it appeared to be an independent prognostic factor [5,6,7,8,9]. However, this negative impact has been controversial in recent trials using different therapeutic combinations and in those previous studies after a longer follow-up [10,11,12,13]. Even though genomic complexity has been mainly defined by the detection of complex karyotypes (CK) by chromosome banding analysis (CBA), genomic microarrays (GM) are also a valuable tool to assess genomic complexity in CLL [14,15]. In addition, optical genome mapping (OGM) has arisen as a promising cytogenomic methodology for whole genome screening, able to detect all types of structural and copy number alterations (CNA) at a higher resolution than traditional cytogenetic methods. Recently, several groups have proven that OGM is a useful technique to detect a wide range of clinically significant cytogenomic abnormalities in different hematological neoplasms [16,17,18,19].

The emergence of GM and other high-resolution molecular techniques, such as next-generation sequencing, has allowed the identification of massive genomic alterations characterized by the occurrence of multiple genomic rearrangements, often generated in a single catastrophic event. These processes are globally referred to as chromoanagenesis and include chromothripsis, chromoanasynthesis and chromoplexy [20,21]. Chromothripsis (cth) (Greek, “chromo” for chromosome; “thripsis” for shattering into pieces) is a unique catastrophic event in which tens to hundreds of genomic fragments are shattered and randomly stitched together due to the subsequent erroneous repair mechanisms, producing highly derivative chromosomes. This process was initially described in a CLL patient as the presence of ≥10 oscillating switches between two or three copy number states in one or a few chromosomes [22]. Nonetheless, some authors also considered those with at least seven copy number switches to be cth events [23,24,25]. Several models have been proposed in order to explain its origin, including chromosome pulverization within a micronucleus, premature chromosome condensation or fragmentation of dicentric chromosomes during breakage–fusion–bridge cycles, among others [26,27,28]. However, the mechanisms underlying the formation of these complex patterns are still unknown. Its prevalence is highly variable and ranges between 2 to almost 100% among different tumors [29,30,31]. In CLL patients, cth prevalence is low (1–3% in unselected cohorts), and most studies are limited to a small number of cases [24,32]. Globally, reported cases present great heterogeneity in terms of the type and number of structural variants but also in the genomic regions and chromosomes affected. Nonetheless, this phenomenon preferentially occurs in certain chromosomes (2, 3, 6, 8, 9, 11, 13 and 17), and some authors have suggested a potential role of genes located in the recurrently abnormal regions in cth development [22,23,24,25,31,32,33,34,35,36,37]. In addition, no detailed comparison between patterns observed in chromothriptic chromosomes detected by GM or NGS and their corresponding karyotype has been performed to date. As for its clinical impact, it has been related to *TP53* abnormalities (found in approximately 70–80%) and a shorter time to first treatment and overall survival [23,24,25,32]. Nevertheless, the assessment of cth is not included in the International Workshop on CLL guidelines [38]. It is noteworthy that although it is known that cth is frequently found in the context of complex genomes, none of the aforementioned studies explored the impact of the overall genomic complexity on the evolution of these cases. In this regard, our group recently reported a strong association between cth and CK and the poor prognosis associated with cth, even within the CK subset [15]. However, the impact of *TP53* status and other clinico-biological characteristics in these patients with cth merits further exploration.

The aim of the present study was to describe the clinical and genomic characteristics of a cohort of 33 CLL patients with patterns of cth detected by GM, especially focusing on the relationship of cth with the overall genomic complexity. Furthermore, we compared cth cases with a cohort of non-chromothriptic CLL cases with CK to elucidate whether the presence of these highly complex patterns could have a negative effect on survival in this particular subgroup. Finally, we analyzed nine cases using OGM to determine the utility of this novel technique in the identification of cth.

## 2. Materials and Methods

### 2.1. Patient Cohort

A total of 162 CLL patients with genomic complexity detected by GM were selected. Among them, 33 showed patterns of cth by GM. The remaining 129 cases, which also showed CK by CBA (≥3 abnormalities in the same cell clone) but did not display cth, were considered as the control group for the comparison of clinical and biological characteristics [15]. All patients had CBA and GM results available at diagnosis or prior to treatment. Demographic, clinical and biological characteristics are summarized in Table 1. Thirty patients with cth and the control cohort were selected from a previous work from our group [15]. The three additional cases with chromothripsis were identified in another study from our group [39].

### 2.2. Genomic Microarray Analyses

DNA was extracted from whole peripheral blood (PB) (n = 7; 21.2%), PB mononuclear cells (PBMCs) (n = 8; 24.2%), PB CD19+ purified cells (n = 13; 39.4%) or from bone marrow samples (n = 5; 15.2%) obtained no more than one year after CBA (median: 0 months; range: 0–12). Only DNA that fulfilled the required quality controls was amplified, labelled and hybridized using different genomic microarray platforms according to the manufacturers’ protocols [ThermoFisher Scientific (n = 25; 75.8%), Agilent (n = 5; 15.2%) and Illumina (n = 3; 9.0%)] (Table S1). The number of abnormalities was recorded as previously described [15]. Chromothripsis was defined by the presence of ≥7 oscillating switches between two or three copy number states on an individual chromosome [23,24,25]. Coordinates were given according to the annotations of genome version GRCh37/hg19.

### 2.3. Optical Genome Mapping

For each sample, a minimum of 1.5 million PBMCs were used to extract ultra-high molecular weight (UHMW) DNA, following the manufacturer’s instructions (Bionano Prep Frozen Cells DNA Isolation Protocol, Bionano Genomics, San Diego, CA, USA). Then, UHMW DNA was enzymatically labeled in a sequence-specific manner using the Bionano Prep Direct Label and Stain (DLS) Protocol (Bionano Genomics). The molecules obtained, labeled around 15 times per 100 Kbp, were cleaned up and loaded onto a Saphyr chip and imaged via the Saphyr instrument (Bionano Genomics). In the chip, molecules were linearized in nanochannels by electrophoresis, and multiple cycles were run to reach an average genome coverage of 300× (approximately 1300 Gb of data per sample). Imaged molecules ≥150 Kbp were analyzed using the rare variant pipeline (RVP) included in Bionano Solve software (v.3.5, Bionano Genomics, San Diego, CA, USA) and visualized in Bionano Access software (v1.6, Bionano Genomics, San Diego, CA, USA). The RVP included two algorithms: a structural variant (SV) analysis, based on the comparison of the labeling pattern against a reference assembly (hg19), and a tool to call large CNA inferred from the coverage of labels detected in each genomic interval. Default recommended confidence scores and an OGM control sample dataset provided by Bionano were used to pre-filter the abnormalities initially called by the software. The cut-off was set at 100 Kbp for SVs and 500 Kbp for CNA. Moreover, OGM results were manually reviewed to merge segmented CNA and discard variants found as benign polymorphisms in the Database of Genomic Variants (http://dgv.tcag.ca/dgv/app/home, accessed on 7 March 2022), SVs found to be duplicated in the results and low-quality translocation calls. Finally, the abnormalities detected by OGM in each patient were recorded and those involving chromothriptic regions were compared to the results previously obtained by GM and CBA techniques.

### 2.4. Whole Chromosome FISH Painting

Whole chromosome FISH painting (WCP) was performed in 6/9 cases analyzed by OGM to validate some selected cytogenomic abnormalities. Whole chromosome painting probes (MetaSystems, Altlussheim, Germany) for chromosomes 1, 2, 3, 6, 8, 9, 11, 13, 15, 17, 19 and Y were used. Chromosomes were counterstained with 4′,6′-diamidino-2-phenylindole (DAPI). FISH signals were observed under a fluorescence microscope in order to confirm or discard novel rearrangements revealed by OGM.

### 2.5. Statistical Analyses

Descriptive statistics were used to provide frequency distributions of discrete variables, while statistical measures were used to provide median values and ranges for quantitative variables. Groups were compared using Chi-square or Fisher exact tests for discrete variables and the Mann–Whitney U test for continuous variables. Time to first treatment (TTFT), the primary end-point of the study, was calculated from the date of cytogenetic study to the date of first treatment or last follow-up, whereas overall survival (OS) was defined from date of cytogenetic study to last follow-up or death. The Kaplan–Meier method was used to estimate the distribution of TTFT and OS. Comparisons among patient subgroups were performed via the Log-rank test. One patient with cth was excluded from survival analyses for having previously received treatment. A multivariate analysis using the Cox proportional hazards regression model was used to assess the independent prognostic impact on TTFT. Statistical analyses were performed using SPSS v.23 software (SPSS Inc., Chicago, IL, USA) and R v3.5.2. *p*-values < 0.05 were considered statistically significant.

## 3. Results

### 3.1. Identification of Chromothripsis Patterns by Genomic Microarrays

A total of 33 patients with cth detected by GM were included. Even though the majority of patients displayed cth in only one chromosome (25/33; 75.6%), eight patients showed complex patterns in several chromosomes (range: 2–4). Among the 46 chromothriptic events detected, 25 (54.3%) included changes that alternated between two copy number states, mostly between one and two copies, resulting in discontinuous deletions of several fragments. In 19/46 (41.3%) events, the oscillations involved both gains and losses, while in 2/46 (4.4%), the rearrangements implied only gains of chromosomal material. Furthermore, these oscillations in the copy number state were located either focally, involving only one chromosome arm, or throughout the whole chromosome (n = 17 and 29, respectively). Interestingly, no differences were observed between the patterns found in those chromothriptic events displaying 7–9 oscillating switches (n = 16) and those with ≥10 switches (n = 30) (Table S2). Cth was found in almost all chromosomes, with the most frequently involved chromosomes being 3, 6 and 13 (five cases each) (Figure S1). Of note, three of the five cases with cth in chromosome 3 carried a deletion of the 3p21.31 locus, which includes the *SETD2*, *CDC25A*, *MAP4*, *FBXW12* and *ATRIP* genes. As for cases with cth in chromosome 6, three of them displayed deletion of 6q21, which includes the *FOXO3a* gene. Regarding cth in chromosome 13, all had the 13q14 CLL common deleted region, which involved the *DLEU1* and *DLEU2* genes as well as the microRNAs miR-16-1 and miR-15a, with several additional deletions throughout the whole chromosome arm (Table S3).

In addition, cth patterns detected by GM were compared to the chromosomal aberrations found in the karyotype to assess whether CBA could suggest the presence of this phenomenon. In most of the chromothriptic events (15/46; 32.6%), the presence of multiple losses detected by GM was reported as monosomies by CBA. Notably, these were accompanied by chromosome markers or additional material of unknown origin, which could explain the apparent loss of the entire chromosome. Moreover, 24/46 (52.2%) of the chromosomes involved had different unbalanced structural aberrations, including unbalanced translocations (12/46; 26.1%), which might suggest the involvement of other chromosomes in the formation of cth, additional material of unknown origin (5/46; 10.9%) and single deletions (7/46; 15.2%). Unexpectedly, seven chromosomes with cth did not show any aberration by CBA. Nonetheless, in one of these cases, the karyotype was normal, suggesting non-division of the tumor clone, while in 6/7 cases, other abnormalities in different chromosomes were detected (Table S4 and Figure S2).

### 3.2. Detection of Different Chromothripsis Patterns by Optical Genome Mapping

**Figure 1:**
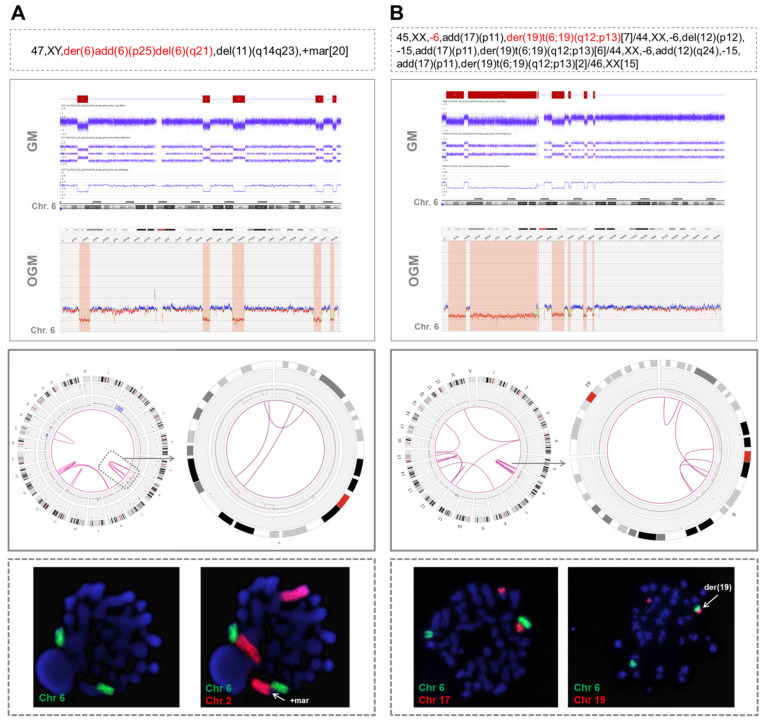
Two examples of cases analyzed by optical genome mapping showing only intra-chromosomal chromothripsis-related rearrangements. (**A**) A patient with chromothripsis in chromosome 6 in which OGM showed the presence of three intra-chromosomal translocations (case #9). When whole chromosome FISH painting (WCP) was performed, two green signals corresponding to chromosome 6 were observed, confirming the presence of material from this chromosome in the one initially reported as “der(6)add(6)(p25)del(6)(q21)” and in its normal counterpart. To ensure that this metaphase was abnormal, chromosome 2 was also stained, since both GM and OGM detected a duplication of 2p. WCP confirmed that the duplicated 2p was the marker chromosome found by CBA. (**B**) Patient with chromothripsis in chromosome 6 in which CBA identified a monosomy 6 and an unbalanced t(6;19)(q12;p13) (case #8). OGM revealed some rearrangements clustered in chromosome 6, but it did not detect this translocation. It was probably not called by OGM due to the involvement of the telomeric region of chromosome 19, a highly repetitive region in which the OGM detection of structural variants is known to be limited. The hybridization pattern obtained by WCP confirmed the presence of this translocation, since two different signals (orange and green), corresponding to both chromosomes, could be observed together but without showing a mixing of these signals, which suggests that they rearranged after the process underlying chromothripsis. The abnormalities detected by CBA in chromosomes with chromothripsis are highlighted in red in the karyotype. Chromosome views show the comparison of the CNA profiles identified by GM and OGM in the chromothriptic chromosomes. The Circos plot represents the abnormalities identified by OGM for the whole genome (on the left) and the chromothriptic chromosome involved in this process (on the right). Different layers show, from outer to inner, cytobands of different chromosomes, structural variants (including deletions, duplications, inversions and insertions), copy number alterations and rearrangements, which are represented by lines joining the chromosomes involved.

Nine patients were analyzed using OGM. This methodology detected almost all the CNA related to cth previously visualized by GM (74/84; 88.1%), showing a high concordance in size and coordinates (Table S5). Remarkably, it also allowed the identification of several rearrangements involving chromothriptic regions (median: 9, range: 3–26), including intra-chromosomal (median: 6, range: 3–12) and inter-chromosomal translocations (median: 5, range: 0–14). Overall, two patterns of rearrangements could be observed. First, in 3/9 cases, the translocations identified by OGM only clustered in the cth region. These findings were in accordance with CBA and FISH results in two of the patients with an abnormal karyotype (cases #8 and #9 in Table S5). In particular, in one patient showing focal cth-related rearrangements on chromosome 6 (case #9), whole chromosome FISH painting (WCP) confirmed the presence of material from this chromosome only in the whole abnormal der(6) and in its normal counterpart (Figure 1A). In the second patient (case #8), OGM rearrangements were also limited to chromosome 6 but CBA identified an unbalanced t(6;19)(q12;p13) that was not detected by OGM, probably due to the limitations of this technique in the detection of abnormalities involving telomeric regions (Figure 1B). Further WCP analyses could not be performed on the third case (case #32), as cells from this patient did not yield abnormal metaphases for CBA. Notably, the three cases with clustered cth-related rearrangements displayed cth in chromosome 6. Notwithstanding, the abnormalities involved were very heterogeneous among these three cases and a commonly deleted region could not be identified. Only small deleted fragments (range: 0.64–2.83 Mb) were common between case #8 and the two remaining cases (cases #9 and #32), with no known gene included (Figure S3).

**Figure 2:**
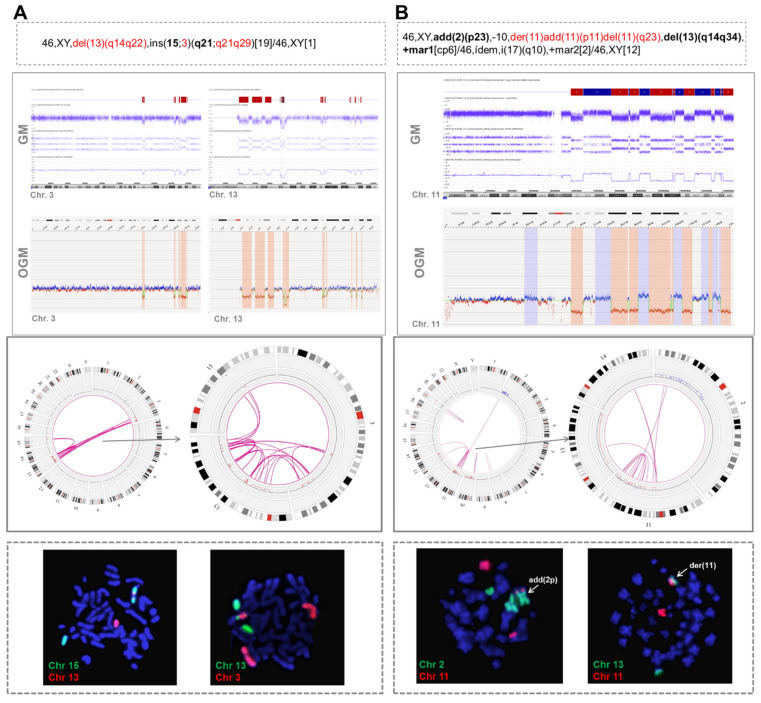
Two examples of cases with chromothripsis analyzed by optical genome mapping showing rearrangements between chromothriptic and non-chromothriptic chromosomes. (**A**) Patient with chromothripsis in chromosomes 3 and 13 (case #31). OGM detected 10 rearrangements between chromosomes 3 and 13 and four rearrangements between chromosomes 13 and 15. Whole chromosome FISH painting (WCP) revealed the presence of material from both chromosomes 3 and 13 inserted in chromosome 15. (**B**) Patient with chromothripsis in chromosome 11 (case #17). OGM revealed the presence of intra-chromosomal translocations and several additional rearrangements involving chromosomes 2, 13 and 14. Notably, t(2;11) and t(11;13) were validated by WCP. Conversely, despite showing only one line in the Circos plot, three parallel t(11;14) were identified by OGM and could not be validated by WCP. However, they could not be ruled out with certainty as true translocations since the rearranged fragment located between the breakpoints was very small and could be missed due to the low resolution of the technique. The abnormalities found by CBA in chromosomes with chromothripsis are highlighted in red in the karyotype. Additional chromosomes associated with chromothriptic events are highlighted in bold. Chromosome views show the comparison of the CNA profiles identified by GM and OGM in the chromothriptic chromosomes. The Circos plot represents the abnormalities identified by OGM for the whole genome (on the left) and for chromothriptic and non-chromothriptic chromosomes involved in this process (on the right). Different layers show, from outer to inner, cytobands of different chromosomes, structural variants (including deletions, duplications, inversions and insertions), copy number alterations and rearrangements, which are represented by lines joining the chromosomes involved.

Second, in 6/9 cases, OGM revealed the presence of rearrangements between the chromothriptic chromosome and other non-chromothriptic chromosomes (Figure 2).

**Figure 3:**
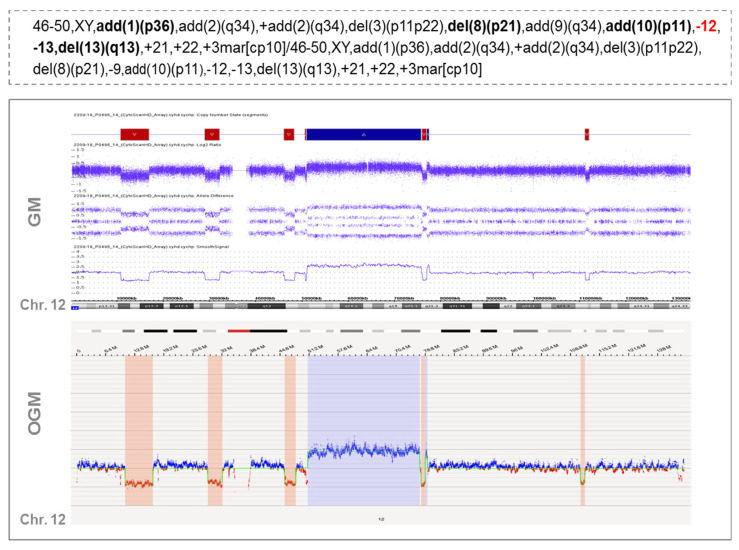

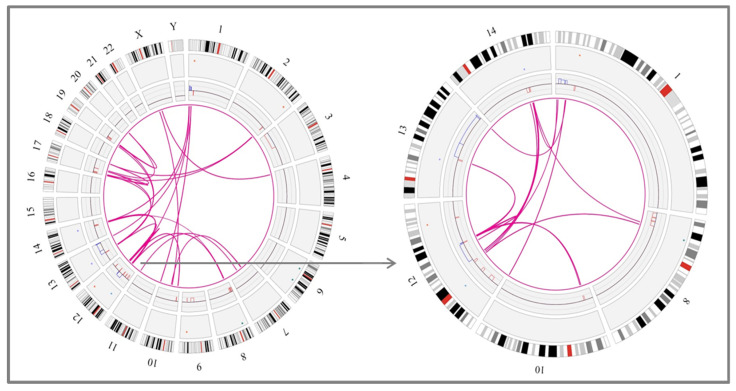
Example of a case with chromothripsis that shows multiple rearrangements with other chromosomes when analyzed by optical genome mapping. Overall, the karyotype of this patient presented a high complexity, with several bits of additional material found in different chromosomes and a few chromosome markers (case #16). Chromosome 12 displayed monosomy by CBA (highlighted in red in the karyotype) but when analyzed by GM and OGM, this chromosome showed an identical pattern of chromothripsis, with >10 switches between 2–3 copy number states. In addition, OGM identified several rearrangements among different chromosomes, as shown in the Circos plot depicted at the bottom of the figure. This highly complex profile could be associated with another catastrophic phenomenon known as chromoplexy, characterized by the presence of multiple chained translocations. Those additional chromosomes associated with chromothriptic events are highlighted in bold in the karyotype.

These rearrangements involved 1 to 6 partners. In one case (case #16), 28 novel translocations were observed along the genome, leading to a very highly complex profile, which could suggest the presence of another catastrophic phenomenon known as chromoplexy (Figure 3). OGM results were compared with CBA data to see whether some of these rearrangements could also be detected in the karyotype. Interestingly, CBA could identify the translocations revealed by OGM in only two cases (cases #2 and #31 in Table S5). Nonetheless, most of the non-chromothriptic chromosomes with novel translocations revealed by OGM were altered, displaying either monosomies together with marker chromosomes or carrying deletions or additional material of unknown origin. Likewise, both GM and OGM also detected CNA in the breakpoints of these non-chromothriptic chromosomes related to chromothriptic events. Several genes were found in the breakpoints. However, none of the novel translocations or genes involved in the breakpoints were common among them (Table S5). Of note, WCP was carried out in six cases in order to validate the rearrangements found by OGM. Most of the novel translocations were confirmed, suggesting the involvement of other chromosomes in the development of cth. Only three new rearrangements could not be validated by WCP (t(11;14) in case #17 in Table S5). However, caution should be taken since the rearranged fragment could be missed due to the limited resolution of the WCP technique (Figure 2B).

### 3.3. Association of Chromothripsis with Other Clinical Characteristics and Prognostic Impact

**Figure 4:**
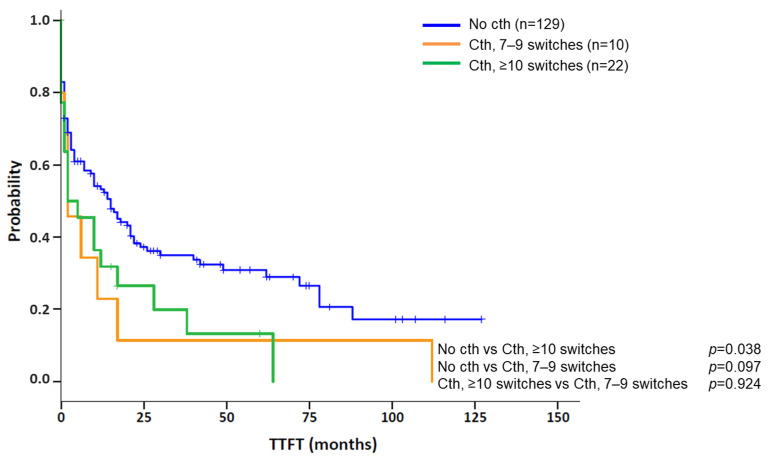
Kaplan–Meier plots for time to first treatment (TTFT) based on the presence of chromothripsis and the number of oscillating switches found in chromothripsis patterns. Kaplan–Meier estimation for TTFT in patients with 7–9 switches between 2–3 copy number states and ≥10 switches between 2–3 copy number states compared to a cohort of CLL cases carrying a complex karyotype (CK) without chromothripsis (cth). Of note, patients were classified into the “Cth ≥ 10 switches” group if they showed at least one chromothripsis event with these characteristics.

Clinical and biological features of patients with cth were compared between the different chromothriptic patterns observed. In the total cohort, 8/33 (24.2%) cases showed cth patterns in more than one chromosome. Among them, the majority (7/8; 87.5%) presented a high complexity, with ≥10 switches in at least one of the chromosomes involved, while only 1/8 (12.5%) displayed patterns with 7–9 switches. Conversely, patients with only one chromothriptic chromosome showed a similar frequency of patterns constituted by 7–9 (10/25; 40%) and ≥10 (15/25; 60%) switches. However, when comparing the abnormalities detected by CBA in both subgroups, no differences could be observed (median: 6 abn. [range: 0–16] in patients with one affected chromosome vs. 7.5 [range: 2–11] in those with >1 chromosome; *p* = 0.481). Likewise, no significant differences could be found among both subgroups in terms of gender, age, IGHV status or frequency of deletions and/or mutations in *TP53* (del/mut*TP53)* or del11q22q23 (*ATM)* (data not shown). Then, in the nine patients studied by OGM, clinical characteristics were compared between patients with only intra-chromosomal cth-related rearrangements and those with involvement of both chromothriptic and non-chromothriptic chromosomes. Overall, both subgroups were similar in gender (66.7% men in both groups), age (median: 67 vs. 66 years; *p* = 0.362), IGHV status (2/2, 100% vs. 5/6, 83.3%; *p* = 1.000) or frequency of del/mut*TP53* (66.7% vs. 50%; *p* = 1.000) or del(11q) (33.3% in both groups). However, the median number of abnormalities was higher in those patients with rearrangements involving non-chromothriptic chromosomes compared with those with clustered rearrangements. These differences were found when the number of abnormalities was recorded both by CBA (median: 8.5 [range: 2–16] vs. 0, 3 and 6 abnormalities, respectively) and GM (median: 19.5 [range: 11–30] vs. 15 [range: 10–15], respectively). Notwithstanding, it is important to note that statistical conclusions cannot be drawn from such a small subset of patients studied by OGM. Next, we aimed to determine whether the number of switches in the copy number state, 7–9 or ≥10, could have different impacts on the outcome of patients with cth. In this regard, patients having only 7–9 switches were compared to those with at least one cth event comprising ≥10. No significant differences were observed for TTFT between these two subgroups (median TTFT: 2 m vs. 2 m; *p* = 0.924) (Figure 4). Therefore, they were considered a unique group for subsequent analyses.

**Figure 5:**
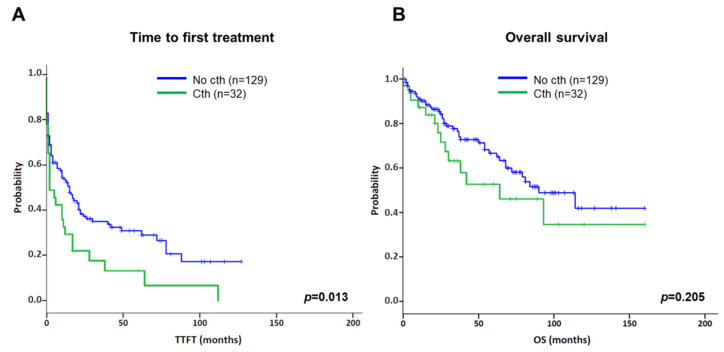
Kaplan–Meier plots for time to first treatment (TTFT) and overall survival (OS) based on the presence of chromothripsis. Kaplan–Meier estimation for TTFT (**A**) and OS (**B**) in patients with chromothripsis (Cth; including cases with 7–9 and ≥10 switches) compared to a cohort of CLL cases carrying complex karyotype (CK) without chromothripsis (No cth).

Concerning the prognostic impact of cth, patients included in the present series were compared to an aggressive cohort of CLL patients carrying CK without cth (Table 1) [15]. In this context, cth had a negative impact on TTFT compared with the control group (median TTFT: 2 m vs. 15 m; *p* = 0.013) (Figure 5A). Likewise, cases with cth showed a tendency towards a shorter OS (median OS: 64 m vs. 90 m; *p* = 0.205) (Figure 5B).

**Figure 6:**
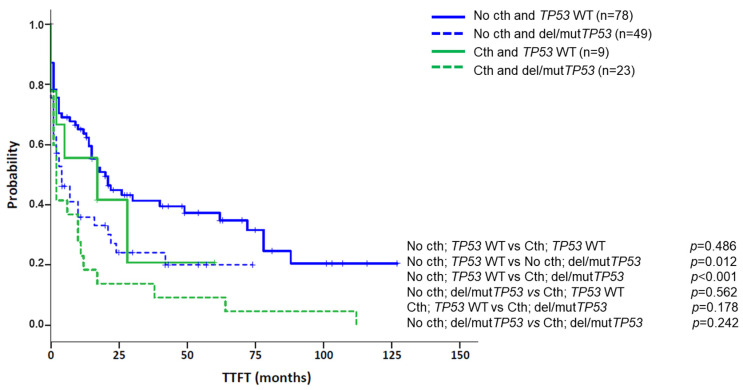
Kaplan–Meier plots for time to first treatment (TTFT) based on the presence of chromothripsis and abnormalities in *TP53* (deletions and/or mutations). Patients were classified according to the presence of chromothripsis (cth) and within each group (No cth vs. Cth), TTFT was assessed based on the presence of aberrations in *TP53* (deletions and/or mutations).

In addition, cth was associated with high genomic complexity and other genomic poor prognostic factors. Particularly, 30/33 patients carried a CK by CBA, with 23 (76.7%) cases having more than five abnormalities. The three non-CK cases by CBA showed genomic complexity as GM identified several abnormalities (median: 10; range: 10–24), with at least three of them ≥5 Mb (range: 3–6). In addition, unmutated IGHV (U-IGHV) and del(11q) were found at frequencies similar to the control group, which is known to be enriched in these poor prognostic markers (74.2% vs. 64.5%, *p* = 0.314, for U-IGHV; 27.3% vs. 32.6%, *p* = 0.560, for del(11q)). In contrast, patients with cth had a higher frequency of del/mut*TP53* than the control group (69.7% vs. 38.6%; *p* = 0.001). Of note, when patients were categorized according to their *TP53* status, cth did not significantly affect TTFT (del/mut*TP53* group, *n* = 72, median TTFT*:* 4 m vs. 2 m, *p* = 0.242, for non-cth and cth, respectively; WT *TP53* group, *n* = 87, median TTFT: 20 m vs. 17 m, *p* = 0.486, for non-cth and cth, respectively) (Figure 6).

On the other hand, no significant differences were observed based on IGHV status or del(11q) for TTFT in the whole cohort (Table 2) and among patients with cth (Cth group, *n* = 30, median TTFT: 1 m vs. 6 m, *p* = 0.412, for mutated-IGHV and U-IGHV, respectively; Cth group, *n* = 32, median TTFT: 6 m vs. 2 m, *p* = 0.079, for non-del(11q) vs. del(11q), respectively). Notably, results from the mutated IGHV subset should be taken with caution since 6/8 cases also carried del/mut*TP53*. Therefore, the tendency towards a shorter TTFT could be attributed to the confounding effects of *TP53*. In the multivariate analysis for TTFT, including genome complexity defined by GM, *TP53* status and cth, only the presence of del/mut*TP53* retained significance (Table 2).

## 4. Discussion

Several works have focused on the study of cth in different types of hematological neoplasms and solid tumors due to its potential role in cancer onset and progression. In CLL, limited numbers of cases have been reported, and they were mainly associated with poor prognostic factors, such as abnormal *TP53* and genomic complexity and a dismal outcome (Table S6). Strikingly, none of the studies published explored the relationship between cth and overall genomic complexity. To the best of our knowledge, this is the largest cohort of CLL with cth assessed in the context of genomic complexity. Herein, an extensive study of the cytogenomic aberrations observed by genomic microarrays was performed, including a comparison with CBA results and an in-depth analysis with novel optical genome mapping technology.

Cth in CLL is highly heterogeneous in terms of the type and number of structural variants, but also in the genomic regions and chromosomes involved [22,23,24,25,31,32,33,34,35,36,37]. In this sense, chromothriptic events described in this cohort mostly involved losses of fragments or alternated losses and gains and were located indistinctly throughout whole chromosomes or focalized in one chromosome arm. The most recurrently involved regions in our series were located at 3p21, 6q21 and 13q14, which had been previously reported in CLL cases with cth [24,25,36]. Regarding the 3p21.31 locus, this region contains the *SETD2* gene, which encodes a histone methyltransferase involved not only in the regulation of gene transcription but also in maintenance of genomic stability, and whose inactivation was also found to be associated with genomic complexity, del/mut*TP53*, cth and aggressive disease [24,36]. Besides, the recurrent deletion affecting the 6q21 locus (containing the *FOXO3a* gene) has been described in 6% of CLL patients and associated with shorter progression-free survival [23,40]. In the context of cth, this region was also deleted in the patient studied by Bassaganyas et al. [34]. The deletion in 13q14, involving the *DLEU1* and *DLEU2* genes as well as the microRNAs miR-16-1 and miR-15a, is the most common cytogenetic lesion in patients with CLL, being present in more than 50% of cases at diagnosis and strongly associated with a favorable prognosis when found as the sole abnormality [41]. However, this good outcome vanishes when accompanied by many other anomalies constituting a pattern of cth [24]. On the other hand, we also found involvement of the previously reported 6p21.1 and 10q24 regions (including *NFKBIE* and *NFKB2*, respectively) in two patients, independently [34]. Likewise, our cohort included one previously reported case with a gain in 5p13.33, which had been suggested as a possible mechanism underlying the formation of these complex rearrangements through the increase in telomerase activity by the deregulation of the *TERT* gene [25]. However, no other case carrying this abnormality was found. Overall, our results confirm that no significant correlation could be established between any of these affected regions and cth onset, suggesting that it might not arise from deregulation of one potential driver gene; rather, it would be initiated by other mechanisms, as yet unknown, that could lead to the alteration of distinct genomic regions.

In the present study, we were able to compare the chromothriptic patterns detected by GM with the chromosomal abnormalities reported in the karyotype. As expected, due to its limited resolution, it was not possible to detect cth by CBA. However, our results confirmed that most of the chromothriptic chromosomes were already altered in the karyotype, carrying a monosomy, deletion or being involved in unbalanced rearrangements. Furthermore, although the chromothriptic chromosomes might display a simple aberration or even be normal, the presence of chromosome markers and additional material of unknown origin was a common feature in these cases, leading overall to more complex karyotypes than those without cth. This was reflected by the higher frequency of cases with ≥5 abnormalities compared with the control group. Thus, CBA can only suggest the presence of complex rearrangements through the detection of abnormalities involving material of unknown origin that might constitute patterns of cth. On the other hand, taking advantage of the structural information provided by the analysis of nine patients using OGM, we were able to reveal rearrangements associated with chromothriptic events, including intra-chromosomal and inter-chromosomal translocations. Even though some of the breakpoints directly overlapped or were close to coding genes, we did not find any common driver gene that could trigger the formation of cth. The novel rearrangements clustered in the chromothriptic chromosomes or involved different non-chromothriptic chromosomes. OGM also underscored a case with a higher complexity profile, comprising several chained translocations involving several chromosomes, which is characteristic of another catastrophic event called chromoplexy. This phenomenon was first identified in prostate cancer, with a prevalence of ~90%, and is also present in other solid tumors [42,43]. However, despite being described in some hematological neoplasms, such as multiple myeloma, mantle cell lymphoma or in previous reports on CLL [24,44,45,46], clinical and biological differences between both chromoplexy and cth and their impact on CLL have not been further explored. In this study, no differences in clinical characteristics could be identified among patients showing the aforementioned rearrangement patterns, although it should be noted that the cohort was too small to draw any conclusion. Even though it is still unknown whether the distinct patterns of rearrangements could affect the evolution of the disease, OGM is a novel technology that provides a more detailed description of these catastrophic events than GM and could shed light on the mechanisms involved in the development of cth. In addition, OGM data analysis is based on a user-friendly interface and could be potentially included in clinical practice. In contrast, even though whole genome sequencing methods have been extensively used for the characterization of cth [22,24,31,34,37], several issues, including high costs and the need for more harmonized analysis pipelines, preclude their incorporation in a routine setting.

Regarding clinical impact, we have shown that CLL patients with cth had a shorter TTFT compared with patients with genomic complexity lacking cth. These results are in accordance with prior studies performed in large CLL cohorts, which reported poor outcomes associated with cth in terms of progression-free survival, TTFT and OS [23,24,25,32]. However, it is noteworthy that the present study only comprised cases with CK and/or genomic complexity detected by GM. Thus, our results should not be extrapolated to a real-life CLL population, where complex cases account for around 10% of patients. Even though cth has been previously associated with genomic complexity, this is the first study focused on this high-risk group. Unfortunately, our series was based on a retrospective and multicenter cohort, including patients receiving different therapeutic agents, which hindered the assessment of overall survival or response to new targeted modalities. As expected, the cth group showed an enrichment of patients with high complexity (≥5 aberrations by CBA) and del/mut*TP53* (both being found in 70% of patients). These frequencies were similar to those defined in previous publications, which, despite not describing results from CBA data, reported high complexity in these cases and a significant association of cth with *TP53* abnormalities (up to 81%) [23,24,25,32]. Notwithstanding, these known independent poor prognostic markers were significantly increased, even when cth patients were compared with the control group with CK, also known to be related to these features. It is therefore conceivable that the poorer evolution observed in cases with cth might be related not only to the chromothriptic event itself but also to the presence of high complexity by CBA or *TP53* abnormalities. In this regard, cth did not affect TTFT when cases were categorized according to *TP53* status. Indeed, in the multivariate analysis, *TP53* was the only parameter maintaining its prognostic significance, reinforcing this hypothesis. Therefore, cth does not represent an independent prognostic factor for CLL patients with genomic complexity, and its identification is not essential in the clinical setting. In summary, a strong connection between *TP53* and cth has been demonstrated. Nonetheless, it would be very interesting to better explore the characteristics and the clinical evolution of cth in cases with wild-type *TP53*, in which other mechanisms would promote the survival of the clones with cth and the confounding effects of *TP53* dysfunction on survival would be avoided.

## 5. Conclusions

In conclusion, chromothripsis is a recurrent event in CLL patients with genomic complexity and is strongly associated with an increased frequency of *TP53* abnormalities. In this sense, the short survival observed in CK cases with cth might actually be due to the increased prevalence of del/mut*TP53*. In addition, OGM has proven to be a valuable cytogenomic tool, capable of detecting previously known abnormalities and identifying new rearrangements, affording an improved view of the highly complex genomic landscape of these patients. However, important questions remain regarding the mechanisms underlying cth and the impact of these patterns on the onset and evolution of CLL. Therefore, further studies with larger cohorts, including more cases with cth and preserved *TP53*, are needed to better understand the role of this phenomenon in CLL pathogenesis and prognosis, particularly in patients treated with new therapeutic agents, to better elucidate the potential of cth as a predictive marker.

## Figures and Tables

**Table 1:** Baseline characteristics of patients at diagnosis and last follow-up.

	Chromothripsis n = 33; n (%)	Control Group n = 129; n (%)	*p*-Value
**Gender**			
Men	23 (69.7%)	93 (72.1%)	0.785
**Median age at diagnosis**	66 years [33–91]	69 years [37–96]	0.177
**Complex karyotype by CBA**	30 (90.9%)	129 (100%)	0.008
3–4 abnormalities	7 (23.3%)	74 (57.4%)	0.001
≥5 abnormalities	23 (76.7%)	55 (42.6%)	
**Stage at diagnosis**			
MBL	1 (3.0%)	1 (0.8%)	0.367
CLL	32 (97.0%)	128 (99.2%)	
Binet A	16/30 (53.3%)	66/109 (60.6%)	0.532
Binet B/C	14/30 (46.7%)	43/109 (39.4%)	
**Common CLL genomic aberrations** *			
del(13)(q14)	19 (57.6%)	80 (62.0%)	0.641
Trisomy 12	1 (3.0%)	26 (20.2%)	0.018
del(11)(q22q23)	9 (27.3%)	42 (32.6%)	0.560
Aberrations in *TP53*	23 (69.7%)	49/127 (38.6%) **	0.001
del(17)(p13)	22 (66.7%)	45 (34.9%)	0.001
*TP53* mutation	13/31 (41.9%)	32/119 (26.9%)	0.104
**Unmutated IGHV**	23/31 (74.2%)	71/110 (64.5%)	0.314
Median follow-up [range] ***	28 months [0–160]	33 months [1–160]	0.490
**Time from diagnosis to cytogenetic study**	1 month [0–298]	0 months [0–129]	0.163
Treatment ***			
Treated patients ^	29 (87.9%)	86 (66.7%)	0.017
Median time to first treatment [95% CI]	2 months [0–6]	15 months [9–21]	0.013
Survival ***			
Median overall survival [95% CI]	64 months [16–112]	90 months [59–121]	0.132

* Deletions and trisomy detected by FISH and/or genomic microarrays. ** Cases in which *TP53* mutation screening was not performed and FISH and/or genomic microarrays were negative for deletion were not considered. *** Data regarding treatment and follow-up from the control group were updated with respect to the previous publication. ^ Patients treated during the follow-up of the study. All samples used for the analysis, except one, were collected prior to treatment. Abbreviations: MBL = monoclonal B-cell lymphocytosis, CI = confidence interval.

**Table 2:** Univariate and multivariate analysis for time to first treatment (TTFT).

Variable	Univariate Analysis *		Multivariate Analysis	
	Median TTFT in Months (95% CI)	*p*-Value	Hazard Ratio (95% CI)	*p*-Value
**GM**				
Intermediate-GC vs. low-GC	21 (4–38) vs. 17 (12–22)	0.941	0.91 (0.53–1.56)	0.719
High-GC vs. low-GC	3 (0–6) vs. 17 (12–22)	0.006	1.45 (0.82–2.57)	0.205
**del/mut*TP53***	3 (1–5)	<0.001	1.60 (1.05–2.43)	0.029
**U-IGHV**	10 (3–17)	0.317	NA	NA
**del(11)(q22q23)**	14 (7–21)	0.614	NA	NA
**Chromothripsis**	2 (0–6)	0.013	1.21 (0.76-1.93)	0.422

* In comparison with the CK cohort extracted from Ramos-Campoy et al., 2022. Abbreviations: GM = genomic microarrays, GC = genomic complexity, low-GC = 0–2 copy number alterations (CNA) detected by genomic microarrays, intermediate-GC = 3–4 CNA, high-GC = ≥5 CNA, U-IGHV = CLL with unmutated IGHV, CI = confidence interval, NA = not assessed.

## Data Availability

Detailed chromosome banding analyses, genomic microarrays and optical genome mapping profiles for selected cases are provided in Supplementary Tables. Please contact either bespinet@psmar.cat or apuiggros@psmar.cat for additional data.

## Supplementary Materials

The following supporting information can be downloaded at: www.mdpi.com/article/10.3390/cancers14153715/s1, Figure S1. Number of cases showing chromothripsis for each chromosome. Chromosomes are represented on the *X*-axis and the total number of cases showing chromothripsis for each chromosome is represented on the *Y*-axis. The chromosomes involved most in the cohort (3, 6 and 13) are highlighted in blue. Figure S2. Copy number profiles of some of the chromothriptic chromosomes. Copy number profiles derived from genomic microarray analyses were available for 18 cases. Figure S3. Chromothripsis detected in chromosome 6 in those cases in which optical genome mapping only revealed intra-chromosomal chromothripsis-related rearrangements. Whole chromosome 6 view of genomic microarray results from cases #8, #9 and #32, which carried intra-chromosomal rearrangements when analyzed by OGM, are represented. Only small deleted fragments were common between case #8 and the other two remaining cases. Specifically, three fragments of 2.83 Mb (10,743,398–13,571,692), 1.20 Mb (16,137,146–17,257,084) and 1.17 Mb (86,948,132–88,115,441) were shared between cases #8 and #9 (highlighted in green), and three fragments of 1.79 Mb (7,651,724–9,438,895), 0.64 Mb (19,953,714–20,591,009) and 1.28 Mb (36,084,473–37,366,801) were common between cases #8 and #32 (highlighted in red). Table S1. Genomic microarray platforms used in this study. Table S2. Patterns of chromothripsis found in cases with 7–9 switches and those with ≥10 switches between 2–3 copy number states. Table S3. Detailed information of the chromothriptic events detected by GM, including the genes involved in the rearrangements (*shown in the excel file attached*). Table S4. Detailed information of the chromothriptic events detected by GM and karyotypes obtained by CBA (*shown in the excel file attached*). Table S5. Results obtained by optical genome mapping in those chromosomes with chromothripsis (*shown in the excel file attached*). Table S6. Review of the literature published about chromothripsis in CLL (*shown in the excel file attached)*.
