# Supplementary material for: TP53 Abnormalities Are Underlying the Poor Outcome Associated with Chromothripsis in Chronic Lymphocytic Leukemia Patients with Complex Karyotype
Source: Cancers (Basel). 2022 Jul 29;14(15):3715. doi: 10.3390/cancers14153715 (PMC9367500; doi:10.3390/cancers14153715)
Supplement: Supplementary file 1 [file cancers-14-03715-s001.zip › Supplementary Figures S1-S3.pdf]

***TP53* abnormalities are underlying the poor outcome associated with chromothripsis in chronic lymphocytic leukemia patients with complex karyotype**

Ramos-Campoy, et al

SUPPLEMENTARY MATERIAL

Supplementary Figures

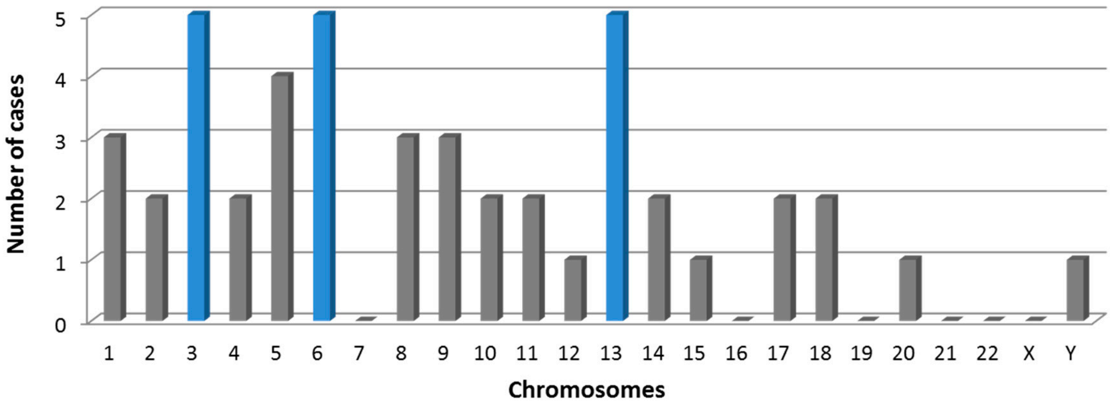

Figure S1. Number of cases showing chromothripsis for each chromosome. Chromosomes are represented on the X-axis and the total number of cases showing chromothripsis for each chromosome is represented on the Y-axis. The chromosomes involved most in the cohort (3, 6 and 13) are highlighted in blue.

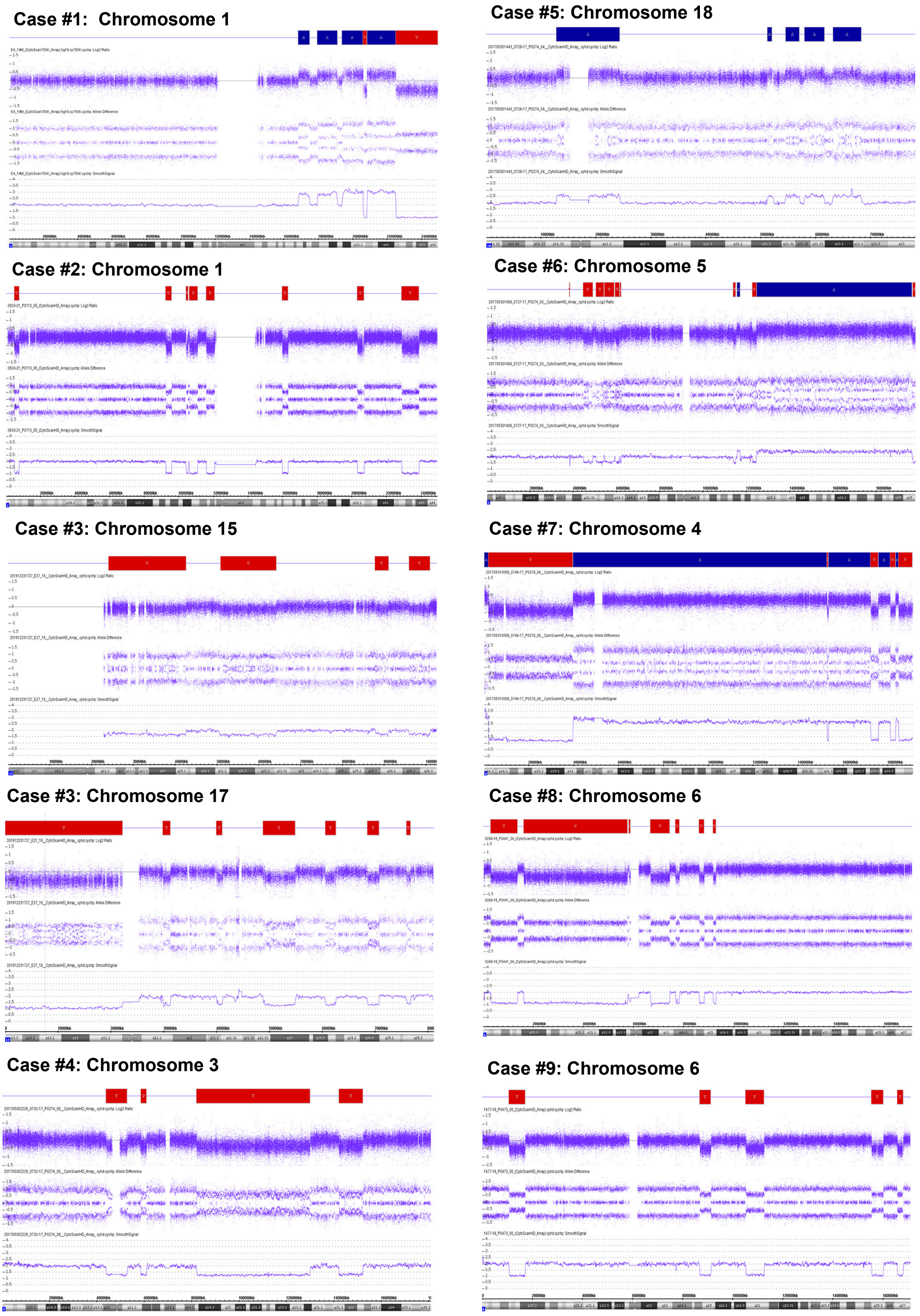

Figure S2. Copy number profiles of some of the chromothriptic chromosomes. Copy number profiles derived from genomic microarray analyses were available for 18 cases.

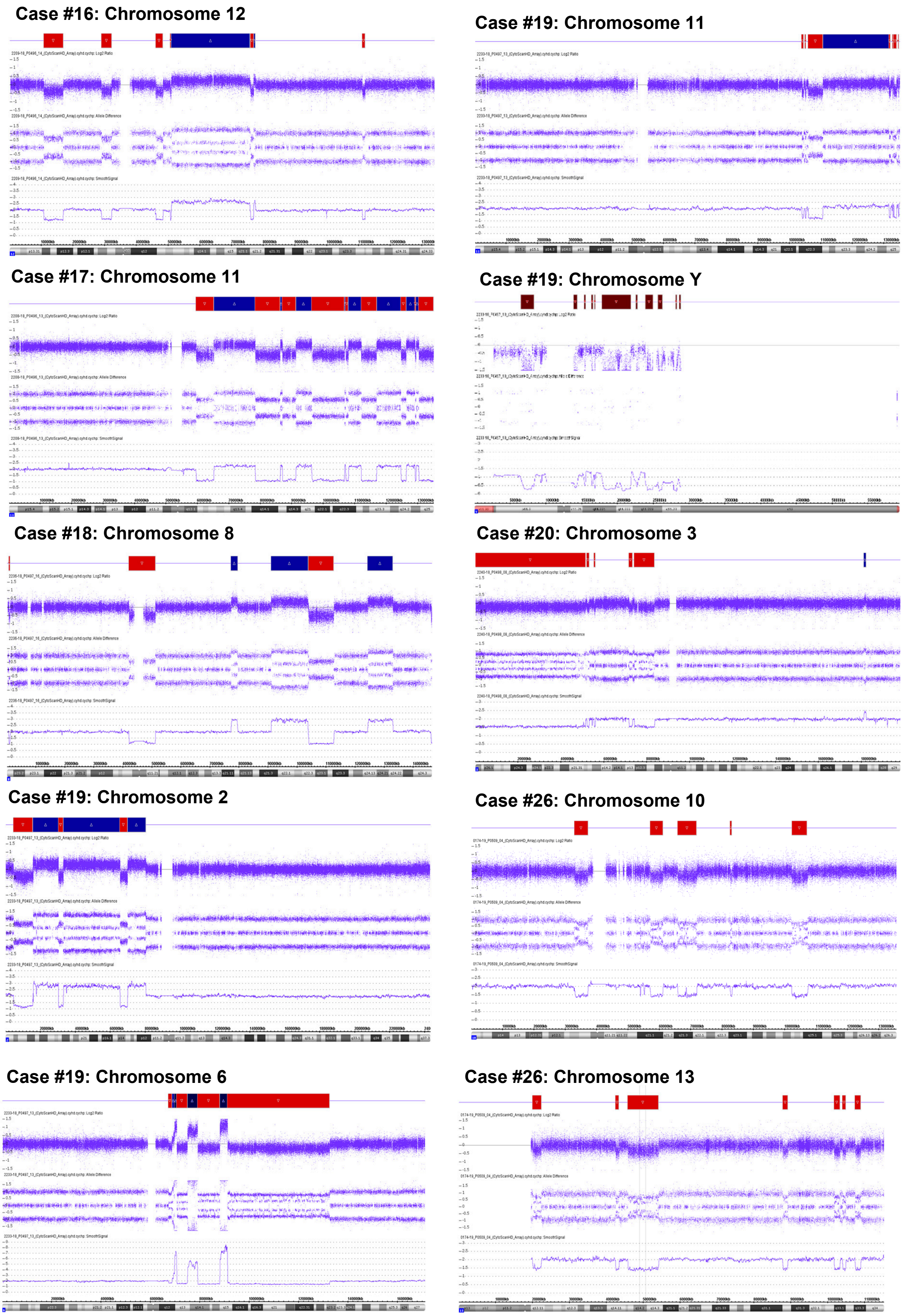

Figure S2 (cont.). Copy number profiles of some of the chromothriptic chromosomes. Copy number profiles derived from genomic microarray analyses were available for 18 cases.

Case #27: Chromosome 6

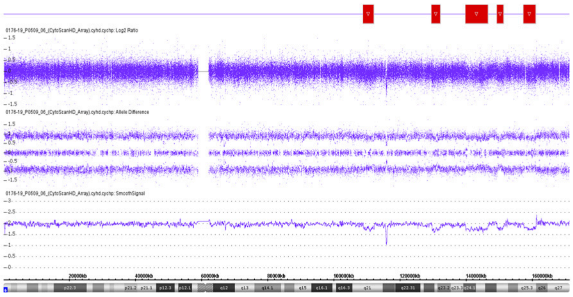

Case #27: Chromosome 14

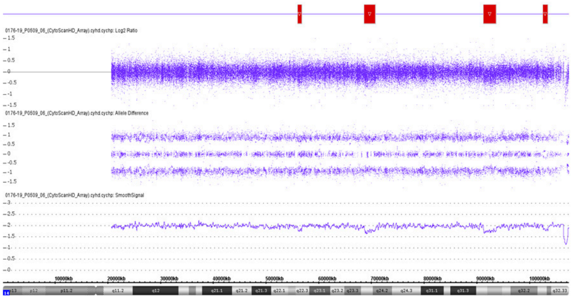

Case #31: Chromosome 3

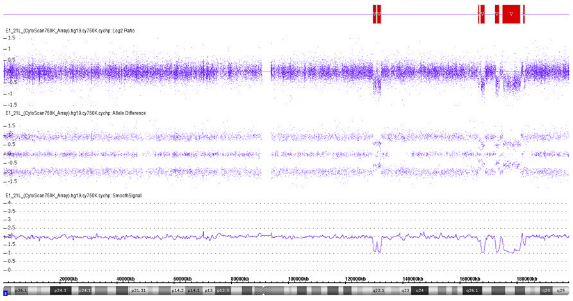

Case #31: Chromosome 13

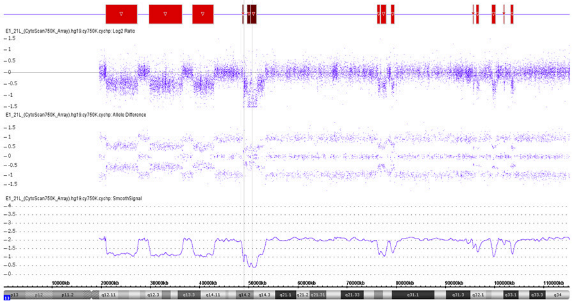

Case #32: Chromosome 6

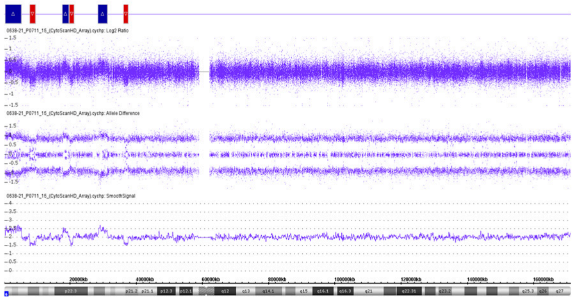

Figure S2 (cont.). Copy number profiles of some of the chromothriptic chromosomes. Copy number profiles derived from genomic microarray analyses were available for 18 cases.

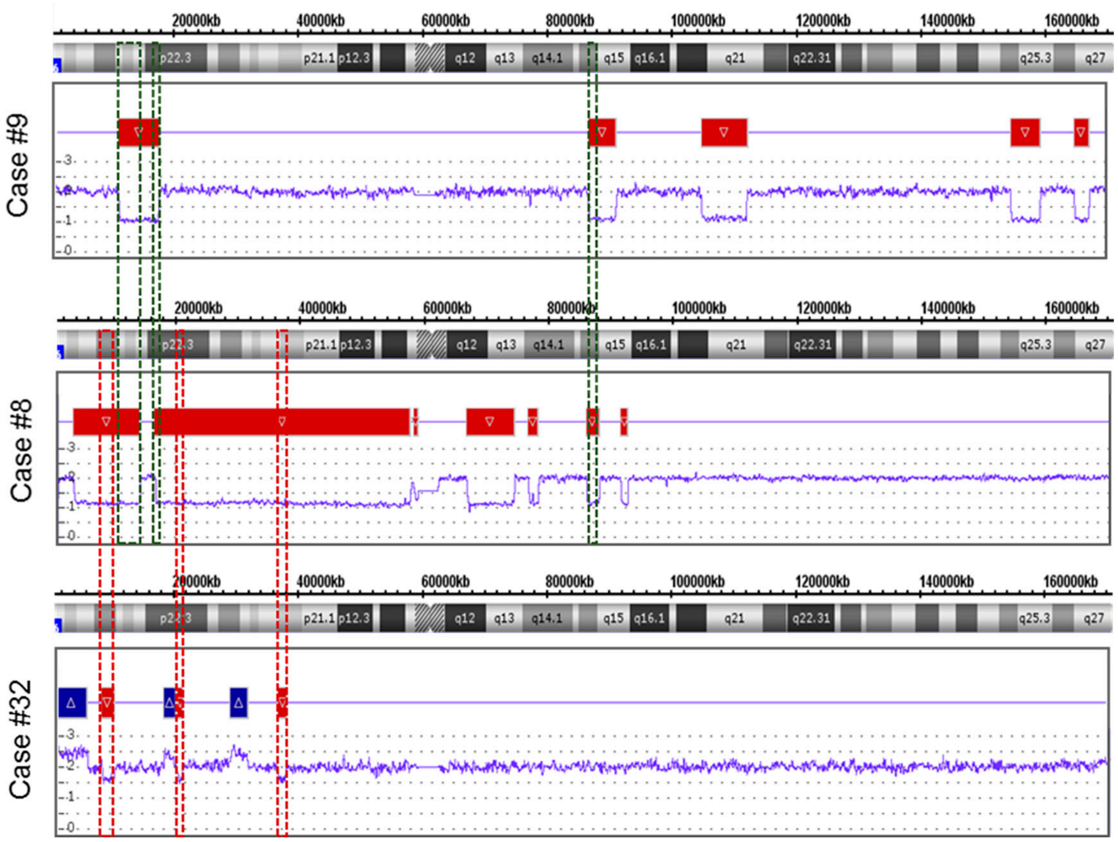

Figure S3. Chromothripsis detected in chromosome 6 in those cases in which optical genome mapping only revealed intra-chromosomal chromothripsis-related rearrangements. Whole chromosome 6 view of genomic microarray results from cases #8, #9 and #32, which carried intra-chromosomal rearrangements when analysed by OGM, are represented. Only small deleted fragments were common between case #8 and the other two remaining cases. Specifically, three fragments of 2.83 Mb (10,743,398-13,571,692), 1.20 Mb (16,137,146-17,257,084) and 1.17 Mb (86,948,132-88,115,441) were shared between cases #8 and #9 (highlighted in green) and three fragments of 1.79 Mb (7,651,724-9,438,895), 0.64 Mb (19,953,714-20,591,009) and 1.28 Mb (36,084,473-37,366,801) were common between cases #8 and #32 (highlighted in red).
