# Supplementary material for: TP53 Abnormalities Are Underlying the Poor Outcome Associated with Chromothripsis in Chronic Lymphocytic Leukemia Patients with Complex Karyotype
Source: Cancers (Basel). 2022 Jul 29;14(15):3715. doi: 10.3390/cancers14153715 (PMC9367500; doi:10.3390/cancers14153715)
Supplement: Supplementary file 1 [file cancers-14-03715-s001.zip › Supplementary Tables S1-S2.pdf]

_TP53_ abnormalities are underlying the poor outcome associated with chromothripsis in chronic lymphocytic leukemia patients with complex karyotype

Ramos-Campoy, et al

SUPPLEMENTARY MATERIAL

Supplementary Tables

Table S1. Genomic microarray platforms used in this study.

Genomic microarray platform	n (%)
**CytoScan 750K array (ThermoFisher)**	2 (6.1%)
**CytoScan HD array (ThermoFisher)**	16 (48.5%)
**Affymetrix SNP6.0 (ThermoFisher)**	7 (21.2%)
**SurePrint G3 Human CGH 8x60K (Agilent)***	1 (3.0%)
**SurePrint G3 ISCA CGH+SNP Bundle, 4x180K (Agilent)**	4 (12.1%)
**Illumina Human Omni1-Quad array (Illumina)**	3 (9.1%)

*Custom design described in Salaverria I, Martín-Garcia D, López C, et al. Detection of chromothripsis-like patterns with a custom array platform for chronic lymphocytic leukemia. Genes Chromosomes Cancer. 2015;54(11):668-80.

Table S2. Patterns of chromothripsis found in cases with 7-9 switches and those with ≥10 switches between 2-3 copy number states.

	**Events with 7-9 switches** n = 16	**Events with ≥10 switches** n = 30	***P-value***
**Type of CNVs**			
Only losses of material	8 (50.0%)	17 (56.7%)	
Only gains of material	1 (6.2%)	1 (3.3%)	0.850
Alternating gains and losses	7 (43.8%)	12 (40.0%)	
**Location of the CNVs**			
Chromosome arm	8 (50.0%)	9 (30.0%)	0.181
Whole chromosome	8 (50.0%)	21 (70.0%)	

Table S3. Detailed information of the chromothriptic events detected by GM including the genes involved in the rearrangements (*shown in the excel file attached*).

Table S4. Detailed information of the chromothriptic events detected by GM and karyotypes obtained by CBA (*shown in the excel file attached*).

Table S5. Results obtained by optical genome mapping in those chromosomes with chromothripsis (*shown in the excel file attached*).

Table S6. Review of the literature published about chromothripsis in CLL (*shown in the excel file attached*).

Supplementary Figures

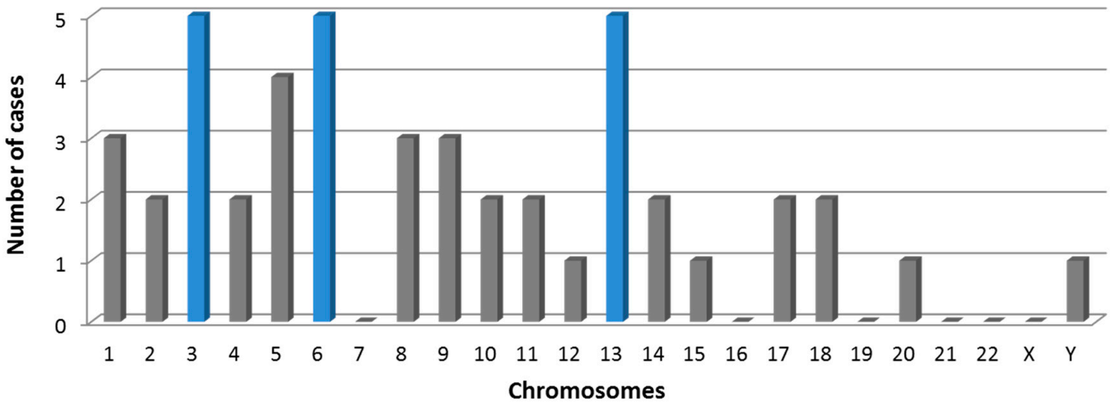

Figure S1. **Number of cases showing chromothripsis for each chromosome**. Chromosomes are represented on the X-axis and the total number of cases showing chromothripsis for each chromosome is represented on the Y-axis. The chromosomes involved most in the cohort (3, 6 and 13) are highlighted in blue.

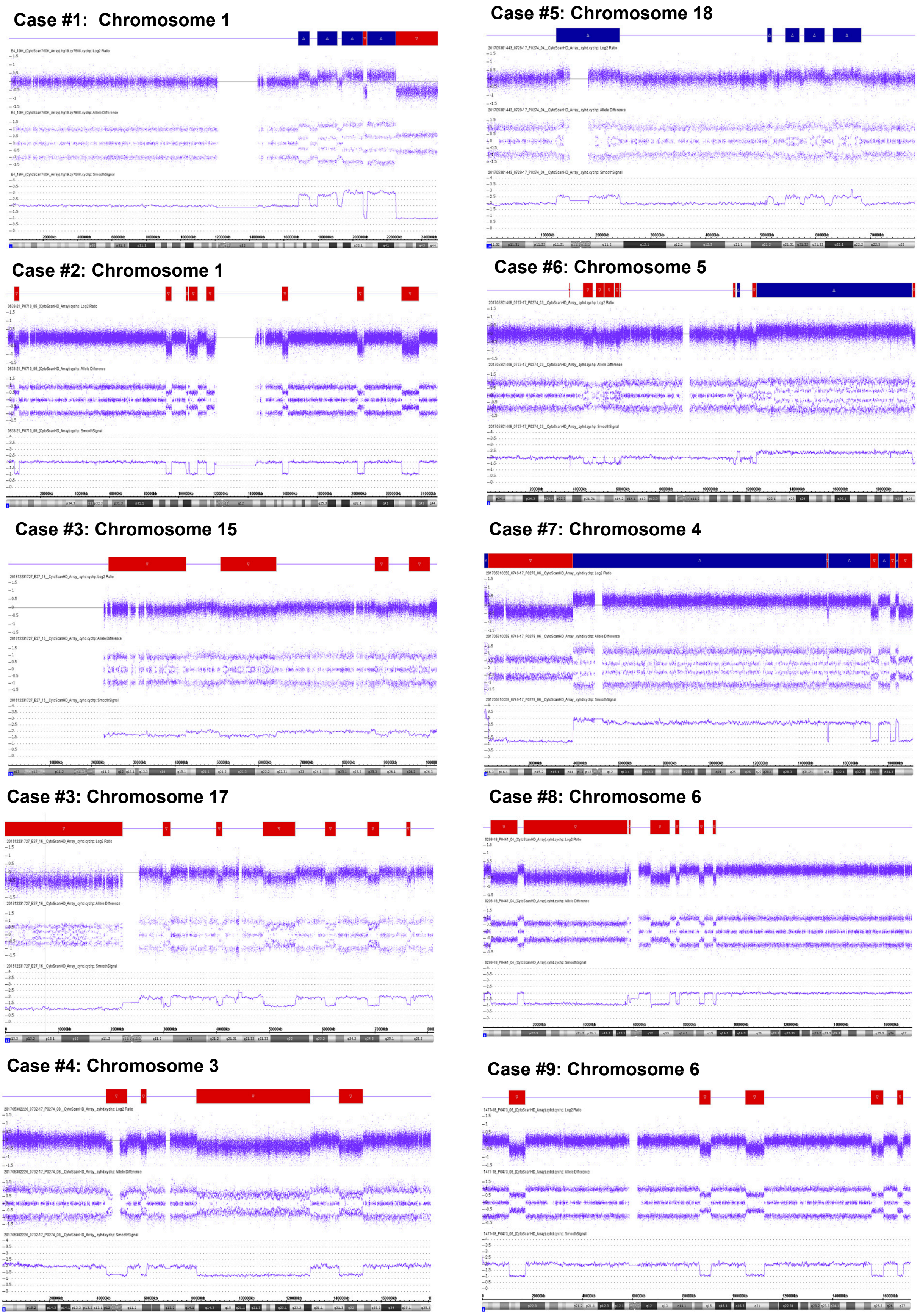

Figure S2. Copy number profiles of some of the chromothriptic chromosomes. Copy number profiles derived from genomic microarray analyses were available for 18 cases.

Case #16: Chromosome 12

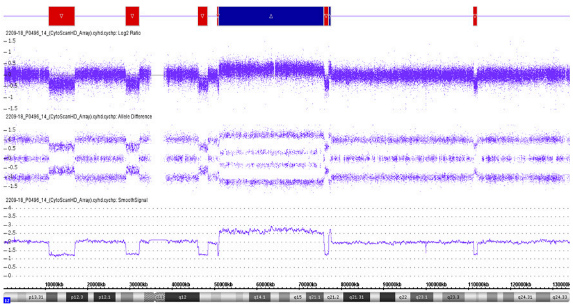

Case #19: Chromosome 11

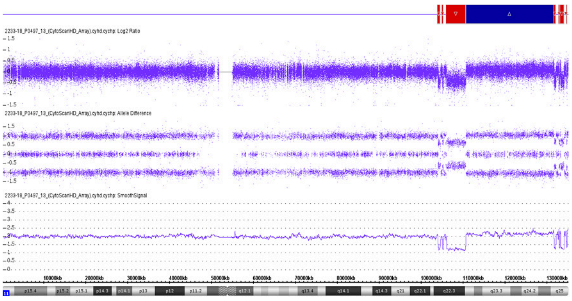

Case #17: Chromosome 11

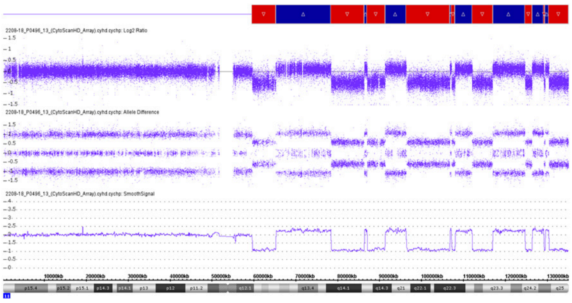

Case #19: Chromosome Y

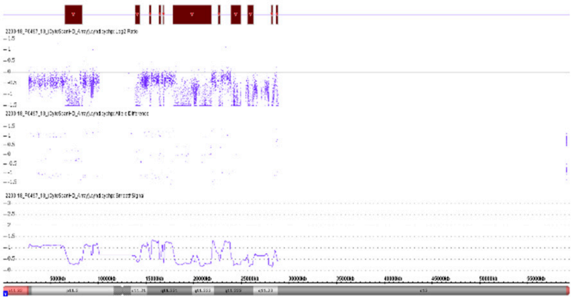

Case #18: Chromosome 8

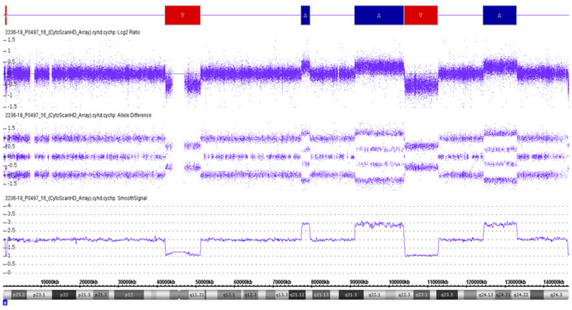

Case #20: Chromosome 3

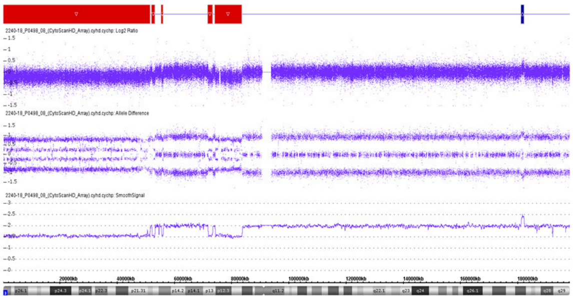

Case #19: Chromosome 2

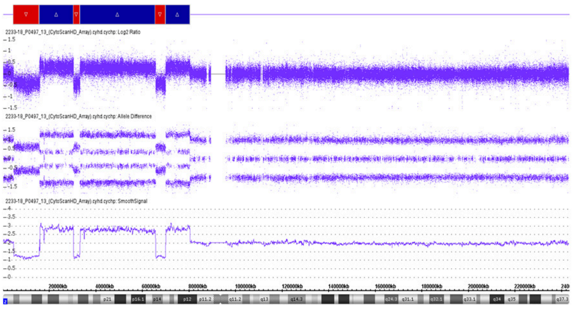

Case #26: Chromosome 10

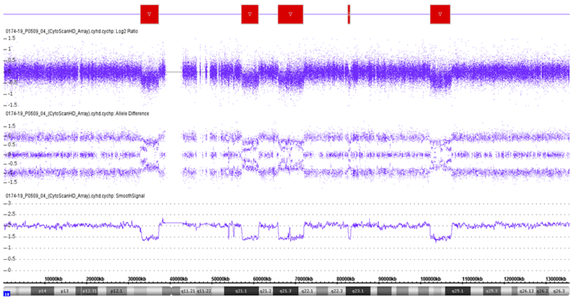

Case #19: Chromosome 6

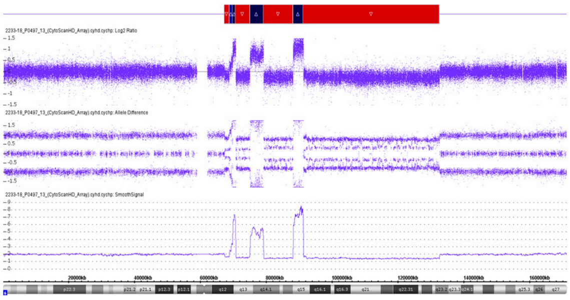

Case #26: Chromosome 13

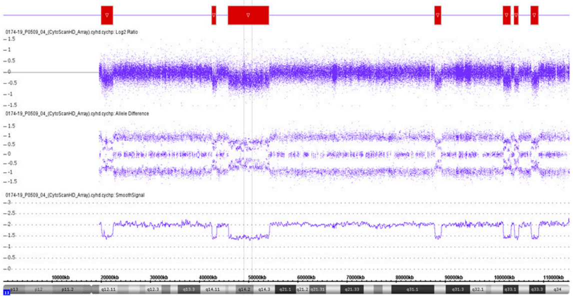

Figure S2 (cont.). Copy number profiles of some of the chromothriptic chromosomes. Copy number profiles derived from genomic microarray analyses were available for 18 cases.

Case #27: Chromosome 6

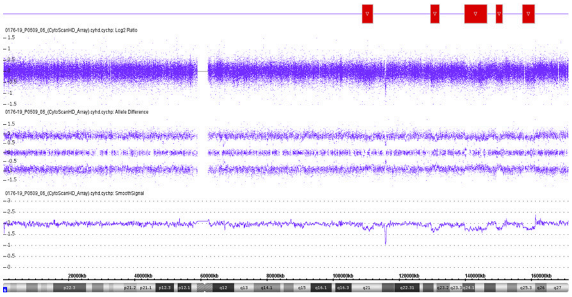

Case #27: Chromosome 14

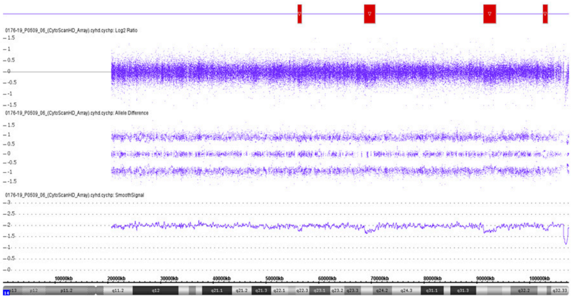

Case #31: Chromosome 3

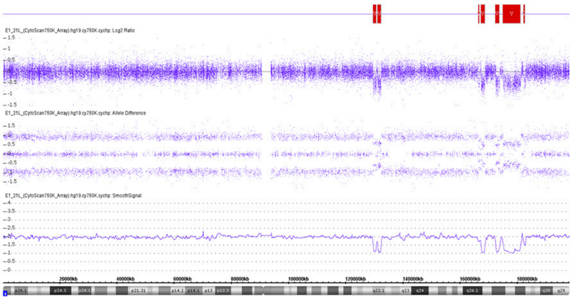

Case #31: Chromosome 13

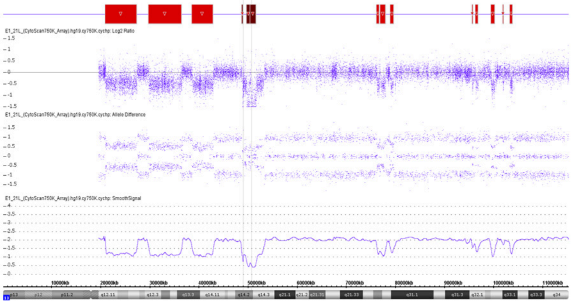

Case #32: Chromosome 6

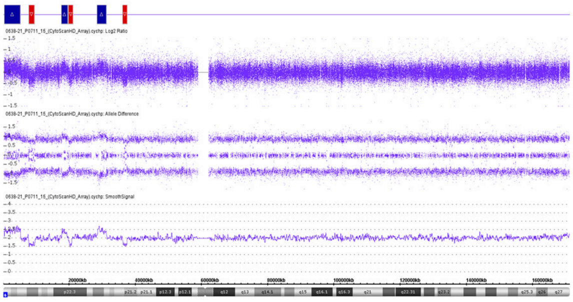

Figure S2 (cont.). Copy number profiles of some of the chromothriptic chromosomes. Copy number profiles derived from genomic microarray analyses were available for 18 cases.

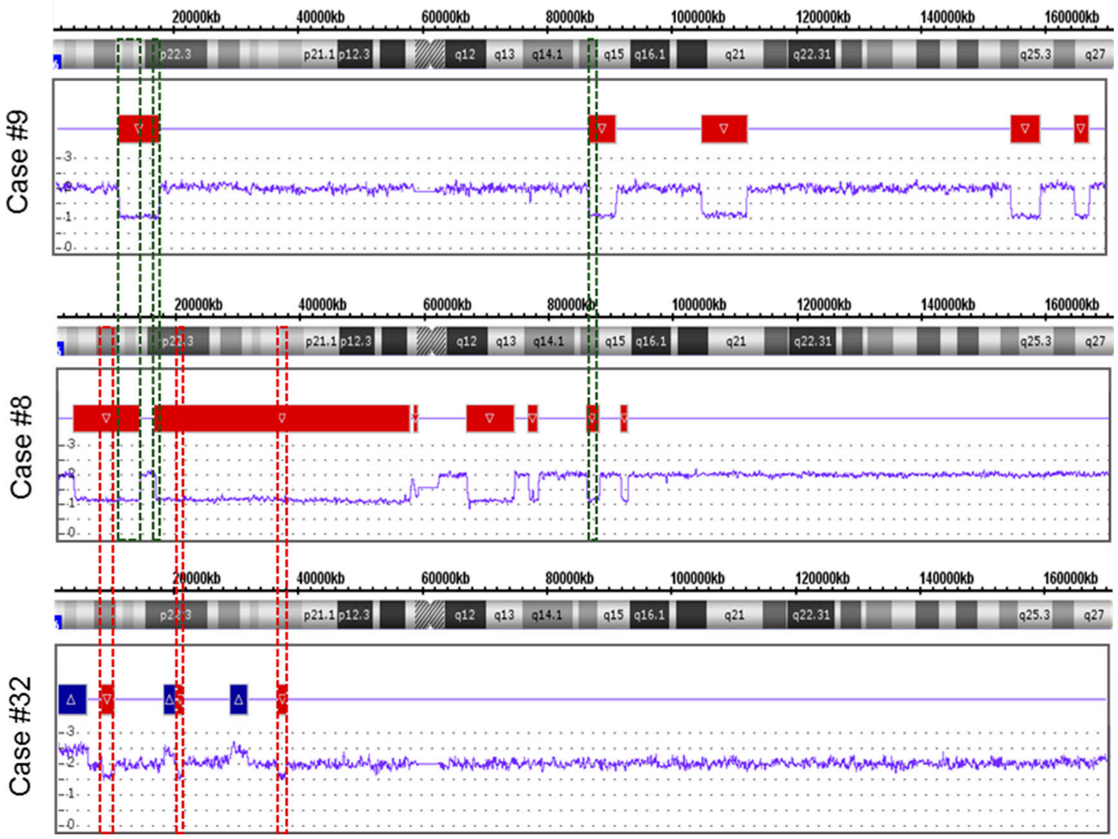

Figure S3. Chromothripsis detected in chromosome 6 in those cases in which optical genome mapping only revealed intra-chromosomal chromothripsis-related rearrangements. Whole chromosome 6 view of genomic microarray results from cases #8, #9 and #32, which carried intra-chromosomal rearrangements when analysed by OGM, are represented. Only small deleted fragments were common between case #8 and the other two remaining cases. Specifically, three fragments of 2.83 Mb (10,743,398-13,571,692), 1.20 Mb (16,137,146-17,257,084) and 1.17 Mb (86,948,132-88,115,441) were shared between cases #8 and #9 (highlighted in green) and three fragments of 1.79 Mb (7,651,724-9,438,895), 0.64 Mb (19,953,714-20,591,009) and 1.28 Mb (36,084,473-37,366,801) were common between cases #8 and #32 (highlighted in red).
